# Identifying Preoperative Predictors for 24-Hour Discharge After Elective Hip and Knee Arthroplasties

**DOI:** 10.7759/cureus.50989

**Published:** 2023-12-23

**Authors:** James Murrell, Nikhil Aravind Khadabadi, Thomas Moores, Fahad Hossain

**Affiliations:** 1 Trauma and Orthopaedics, Walsall Manor Hospital, Walsall, GBR

**Keywords:** hip and knee arthroplasty, hip and knee replacement, complications, discharge, pre-operative predictors, day-case, outpatient, knee arthroplasty, hip arthroplasty, arthroplasty

## Abstract

Introduction

The resumption of elective medical services post-pandemic has brought to the forefront the importance of outpatient arthroplasty services in promoting efficiency and mitigating hospital-acquired infections. This study aimed to identify preoperative factors that predict the success of outpatient lower limb arthroplasty surgeries.

Methods

Our investigation involved a retrospective review of 606 patients who underwent elective hip and knee arthroplasty. We documented variables such as the hospital length of stay, patient demographics (age and gender), Oxford Joint Scores, body mass index, socioeconomic status, American Society of Anaesthesiologists' (ASA) physical status classification, comorbid conditions, the Functional Comorbidity Index (FCI), preoperative blood test results, implant types, scheduling details of the surgery, and rates of readmission within 30 days post-surgery. A two-step analysis using univariate and multivariate regression models was performed to pinpoint preoperative indicators that could predict same-day discharge following arthroplasty.

Results

Forty-five patients (7.4%) were discharged within 24 hours of surgery. Early discharge did not correlate with higher rates of readmission within 30 days (p>0.05). Neither weekend nor afternoon surgeries significantly extended the length of stay beyond 24 hours (p>0.05). No significant differences in the prevalence of comorbidities, FCI scores, socioeconomic status, or preoperative blood test results were found when comparing patients discharged within 24 hours to those who stayed longer. Multivariate analysis revealed that patients younger than 65 years (relative risk (RR) 2.41; 95% confidence interval (CI) 1.02-5.74) and those receiving partial knee arthroplasty (RR 8.91; 95% CI 3.05-26.04) were more likely to be discharged within 24 hours.

Conclusions

Outpatient arthroplasty is a viable option, especially for individuals younger than 65 years undergoing partial knee arthroplasty, independent of other patient-related factors, comorbidities, and specifics of the hospital episode.

## Introduction

The demand for lower limb arthroplasties has substantially increased, culminating in a significant number performed annually in the United Kingdom [1, 2]. The National Health Service (NHS) has encountered considerable strain in elective services following the coronavirus disease 2019 (COVID-19) pandemic, with elective waiting lists in England expanding notably [3]. The elective arthroplasty services have been especially impacted, with record numbers of patients facing extended waits for surgery [3]. A considerable proportion of patients awaiting hip and knee arthroplasty rate their quality of life as extremely poor, with some equating their suffering to being worse than death, a sentiment exacerbated by the prolonged waiting times during the pandemic [4].

The median weighted cost for a primary total hip arthroplasty (THA) is £7,431 [5], and for total knee arthroplasty (TKA), it is £8,392 [6], contributing to an annual expense of over £1.3 billion for the NHS. Hospital stay duration is a critical determinant of the cost associated with arthroplasty [7]. The average length of stay has significantly decreased over the past decades, signalling an improvement in healthcare efficiency [8]. The NHS reports a current median stay of 3.2 days for a primary THA and 3.3 days for a primary TKA procedure [9]. The NHS elective recovery plan, aiming to address the backlog of elective surgeries, includes initiatives to shorten post-arthroplasty hospital stays. The 'Get It Right First Time' programme and the British Orthopaedic Association have been instrumental in promoting ambulatory care pathways that enable day-case or 24-hour discharge post-arthroplasty [3].

Transitioning to day-case arthroplasty for select patients could result in considerable cost savings and increased hospital capacity. Identifying patients who are candidates for same-day discharge remains a priority, striking a balance between cost-effectiveness and patient safety [10]. Establishing objective preoperative criteria would empower clinicians to anticipate patient outcomes and optimise postoperative care [11]. However, standardised criteria for day-case arthroplasty patient selection are still not established [12]. This study primarily seeks to pinpoint preoperative factors predictive of 24-hour discharge suitability following THA, TKA, and unicompartmental knee arthroplasty (UKA) surgery. Secondary objectives include assessing the incidence of readmissions, reoperations, and complications associated with rapid discharge post-arthroplasty. Moreover, there is a scarcity of large-scale studies evaluating the influence of sociodemographic factors, such as patient social deprivation, on the feasibility of outpatient joint arthroplasty and specifically comparing THA, TKA, and UKA procedures.

## Materials and methods

This research was conducted at the Walsall Manor NHS Trust, Walsall, UK. We conducted a retrospective review of our hospital's electronic patient record database, identifying patients who underwent THA, TKA, and UKA from March 2018 to April 2020. We included all adult elective arthroplasties and excluded revision surgeries and procedures performed concurrently with arthroplasties, such as debridement, implant retention, wound washouts, and surgeries addressing periprosthetic trauma. Patients were stratified into two categories: those discharged within 24 hours and those necessitating longer inpatient care. Outliers with extended lengths of stay of more than 10.5 days were excluded from analysis and were defined using Tukey’s Fences method, namely those above the upper quartile by >1.5 times the interquartile range. These cases were excluded from analysis, as they likely represented patients whose discharge was delayed due to reasons other than the direct impact of the surgery.

A medial parapatellar approach was used for both the UKA and TKA procedures. Within our organisation, three out of four surgeons utilised a modified anterolateral approach, and one surgeon utilised a posterior approach for THA. There was no unified opioid-sparing anaesthetic protocol within the hospital trust at the time of data collection, and tranexamic acid was not routinely given to patients.

Patient demographics collected included age, gender, and government indices of social deprivation, with age further categorised into <55 years, 55 to 65 years, 66 to 75 years, and >75 years. Surgical data encompassed the type of operation, American Society of Anesthesiologists (ASA) grade, timing of the surgery (morning or afternoon), and operation duration. Patient comorbidities were recorded using International Classification of Diseases codes to calculate the Functional Comorbidity Index (FCI) for functional outcome assessment [13]. Additionally, we assessed the impact of cognitive impairment and cancer on the capacity for 24-hour discharge, as these factors are not included in the FCI.

Haemoglobin levels and renal function were evaluated based on chronic kidney disease classifications from the UK Kidney Association guidelines [14], with haemoglobin categorised by levels less than or equal to 13 g/dL [15, 16].

Our primary outcome measure was the length of stay, dichotomized into discharge within and beyond 24 hours. We also examined 30-day readmission rates concerning length of stay.

We conducted a univariate analysis of all variables, contrasting patients discharged within 24 hours against those discharged later. Categorical data underwent chi-squared/Fisher's exact test analysis, and continuous data were evaluated using the independent t/Mann-Whitney U-test. A backward, stepwise iterative binary logistic regression model was subsequently selected from the univariate analysis with a significance threshold of p<0.05, as recommended by established statistical protocols [17]. Through an iterative process of regression, findings were reported as odds ratios (ORs). All statistical analyses were carried out using IBM SPSS Statistics software for Windows, Version 21.0 (Armonk, NY: IBM Corp.), with data in tables presented to three significant figures.

## Results

A total of N=698 patients were identified, of whom N=92 were excluded. The remaining N=606 were included in the analysis, of whom 7.4% (N=45) were discharged within 24 hours. Of these, 332 underwent TKA, 261 underwent THA, and 23 underwent UKA. Among the analysed cohort, 4.6% (12 of 261) of THA patients, 7.5% (25 of 332) of TKA patients, and 34.7% (8 of 23) of UKA patients were discharged within 24 hours.

The average age of patients discharged within 24 hours was 65.7 years (standard deviation (SD) ± 9.55), which was significantly younger than the patients requiring a longer inpatient stay, who had an average age of 69.5 years (SD ±9.59; p=0.036). Gender differences approached significance (p=0.107), as did the ASA grade (p=0.117) when comparing the two groups (Table 1).

**Table 1:** Comparison of patient demographics IQR: interquartile range *: statistically significant

Patient Demographics	≤24 Hour Discharge	≥24 Hour Discharge	P-Value
Age			
<65 years	22	162	.036*
≥65 years	23	399	
Sex			
Male	24	230	.109
Female	21	331	
American School of Anesthesiology Grade			
1-2	41	438	.117
3-4	4	123	
Median social deprivation index (±IQR)	3 (5-9)	3 (5-10)	.512

Surgical timing did not significantly differ between the groups, whether during a weekend versus a weekday (p=0.910) or in the morning versus the afternoon (p=0.531). However, the type of operation was a significant factor, with UKA patients more likely to be discharged within 24 hours (p<.001). Additionally, the mean operative time was shorter for the <24-hour discharge group at 100 minutes (SD ±24.2) versus 111 minutes (SD ±22.3) for those with a longer inpatient stay (p=0.003).

Preoperative bloodwork revealed that patients discharged within 24 hours had a higher average preoperative haemoglobin level (140 g/L, SD ±14.3) compared to those with extended inpatient admission (135 g/L, SD ±12.9), although this finding showed only a weak statistical correlation (p=0.059). Chronic kidney disease stage did not significantly affect the likelihood of day-case arthroplasty (p=0.458). Individual comorbidity analysis and cumulative index analysis utilising the FCI did not significantly differ between the two groups, with full results and corresponding p-values presented in Table 2.

**Table 2:** Comparison of hospital, surgical-related variables, and comorbidity analysis THA: total hip arthroplasty; TKA: total knee arthroplasty; UKA: unicompartmental knee arthroplasty; SD: standard deviation; Hb: haemoglobin; CKD: chronic kidney disease; COPD: chronic obstructive pulmonary disease; MI: myocardial infarction; GI: gastrointestinal *: statistically significant ^a^: p-value from Mann-Whitney U test as the factor is ordinal

Variables		≤24 Hour Discharge	≥24 Hour Discharge	P-Value
Surgical Variables/Operation Type, n	THA	12	249	< .0001*
	TKA	25	297	
	UKA	8	15	
Hospital Variables, n	Weekday	31	391	.910
	Weekend	14	170	
	AM	21	289	.531
	PM	24	272	
Patient's Preoperative Bloodwork	Mean Hb Level (±SD), g/dL	140 (± 14.3)	135 (± 12.9)	.050*
Chronic Kidney Disease, n	CKD 0-1	41	476	.513
	CKD 2-3	4	85	
	CKD 4-5	0	1	
Functional Comorbidity Index (FCI)	FCI 0	0	11	.303^a^
	FCI 1	14	142	
	FCI 2	17	181	
	FCI 3	8	130	
	FCI 4	5	64	
	FCI ≥ 5	1	33	
Comorbidity Analysis, n (%)	Arthritis	44 (98)	542 (97)	.674
	Osteoporosis	1 (2)	9 (2)	.754
	Asthma/COPD	7 (16)	105 (19)	.599
	Angina	7 (16)	76 (14)	.706
	Heart Failure	0 (0)	5 (1)	.525
	MI	3 (7)	60 (11)	.394
	Neurological Disease	4 (9)	72 (12)	.442
	Stroke	0 (0)	1 (0)	.777
	Diabetes	6 (13)	95 (17)	.533
	GI Disease	7 (16)	105 (18)	.599
	Depression/Anxiety Disorders	9 (20)	112 (20)	.995
	Sensory Impairment	1 (2)	16 (3)	.806
	Degenerative Disc Disease	0 (0)	5 (1)	.525
	Obesity	12 (27)	150 (3)	.902
	Malignancy	4 (9)	43 (8)	.768
	Cognitive Impairment	1 (2)	10 (2)	.832

Additionally, 30-day readmission and mortality rates were not significantly different between patients discharged within 24 hours and those discharged after 24 hours (p=0.366; Table 3).

**Table 3:** Thirty-day readmission comparison

30-Day Readmission	≤24 Hour Discharge	≥24 Hour Discharge	P-Value
Yes, n	0	10	.366
No, n	45	551	

A multivariate Cox regression analysis included the following variables: age, type of procedure, and preoperative anaemia. The analysis revealed that being younger than 65 years (relative risk (RR) 2.41, 95% confidence interval (CI) 1.02-5.74) and undergoing UKA (RR 8.91, 95% CI 3.05-26.04) were statistically significant predictors of discharge within 24 hours. These results are summarised in Table 4.

**Table 4:** Summary of the Cox multivariate regression analysis TKA: total knee arthroplasty; UKA: unicompartmental knee arthroplasty; Hb: haemoglobin

Variable		Relative Risk	95% Confidence Interval
Age	55-64 years	2.41	1.02-5.74
	Age ≥65 years	1.32	0.55-3.167
Type of Procedure	UKA	8.91	3.05-26.04.
	TKA	1.794	0.877- 3.67
Anemia	Preoperative Hb <13 g/dL	1.716	0.814- 3.616

## Discussion

The feasibility of day-case arthroplasties is becoming apparent, with patient selection and the development of structured pathways within orthopaedic departments enhancing efficiency [7, 18]. This is particularly pertinent for the NHS, as it seeks to curtail unnecessary expenditures [19] and mitigate the risk of COVID-19 among elective patients.

Our study identified two predictors for successful discharge within 24 hours: patients under 65 years of age and those undergoing UKA. Supporting evidence indicates that a younger age significantly enables same-day discharge for UKAs [20, 21]. Furthermore, the literature identifies being over 80 years old as a risk factor for complications post-joint arthroplasty [10, 22].

We observed that the mean operative time was significantly shorter for patients discharged within 24 hours (100 vs. 111 minutes; p=.003). However, this variable was not included in the regression analysis, as it does not serve as a preoperative predictor despite being an independent variable for achieving day-case arthroplasty. This may reflect the shorter operative time for UKA vs. TKA procedures, with one study finding that the operative time was significantly longer for TKA than for UKA (112 minutes vs. 81 minutes)[23,24].

Previous studies reported that same-day discharge for total hip and knee arthroplasties is both safe and effective [25-30]. In contrast, a large multicenter study from the United States reported same-day discharge rates of 3.9% for THA and 2.3% for TKAs out of 120,847 patients [10]. Our study reported similar rates for THAs and a higher percentage for TKAs. Patient concerns about same-day discharge after arthroplasty include difficulties with bathroom access, pain management, fall risk, and a lack of home support despite recognising the benefits [31].

A national study from the United States, including over 50,000 total knee and hip arthroplasties, indicated that early discharge was not associated with increased 30-day complications or readmissions [32]. Our findings corroborate this. Interestingly, a study by Gabor et al. reported lower 90-day readmission rates for day-case total hip replacements compared to inpatient procedures [33]. Our study concurs, showing no significant difference in 30-day readmission rates (p=0.366).

In our research, neither ASA grades nor patients' comorbidities significantly influenced the likelihood of 24-hour discharge. This finding is at odds with some studies that suggest ASA grades correlate with length of stay and success rates of same-day discharge [10, 34, 35]. In contrast, we used the FCI, which has been shown to predict physical function more accurately [13].

Conversely, Bradley et al. found that ASA grades did not affect the success of same-day discharge for patients undergoing UKAs [20]. Regarding comorbidities, a systematic review highlighted that patients discharged within 24 hours typically have fewer functional dependencies and chronic conditions [10].

Several scoring systems are employed to predict postoperative outcomes, such as the Rapid Assessment and Prediction Tool [36] and the Blaylock Risk Assessment Screening Score [37]. These tools are designed to identify the risks of complications and the need for prolonged rehabilitation rather than to predict same-day discharge [11, 38]. Our findings suggest that patient age and UKA are sufficient indicators for same-day discharge, a conclusion not supported by including comorbidities in the Blaylock score. Moreover, ASA grades and the Charlson Comorbidity Index are unreliable predictors of same-day discharge capability [39].

The Outpatient Arthroplasty Risk Assessment Score demonstrates efficacy in identifying candidates for same-day discharge but is less effective for those who are not. A review of over 1,000 cases showed that the score falsely identified 97% of patients as unsuitable for same-day discharge [40], indicating the need for more refined assessment tools. Our study demonstrates that social deprivation does not significantly influence the likelihood of outpatient arthroplasty success (p=0.512), challenging current assertions that socioeconomic status correlates with extended hospital stays post-lower limb arthroplasty [34, 41].

The work of Keulen et al. indicates that patients undergoing UKA are more likely to be discharged on the same day than those receiving TKA. This may be due to UKA preserving knee joint kinematics and requiring less bone removal, along with a less invasive approach [35]. The biomechanical benefits of UKA, as UK studies suggest, include fewer serious medical complications, lower mortality, quicker recovery, and better functional outcomes given an appropriate patient population [42-44]. Additionally, UKA is associated with fewer complications, notably pain [45]. Drager et al. found that UKA patients had a median hospital stay of two days compared to three for TKA patients [46], which aligns with our findings of an average 2.19-day stay for UKA. These results support the feasibility of sub-24-hour hospital admissions through targeted enhanced recovery pathways emphasising early mobility and pain management [47].

Comparative research shows no significant difference in the time to independent walking, knee flexion, or knee-specific patient-reported outcomes, such as the Oxford Knee Score (OKS), between TKA and UKA up to three years post-operation [24, 48, 49]. An extensive study with 38,000 participants revealed six-month postoperative OKS averaging 38 (SD 9.4) for UKA versus 36 (SD 9.4) for TKA. The UKA patients were likelier to achieve excellent OKS (≥ 41) at 47% compared to 36% for TKA patients, and fewer had poor OKS (< 27), at 13% versus 16%. Moreover, the EuroQol 5-Dimension 5-Level quality of life metric showed greater improvement following UKA than TKA [50].

An independent analysis within the UK NHS posits that UKA is cost-effective, functionally superior, and underused [19]. Systematic reviews have identified that single medial compartment osteoarthritis is most common, suggesting a significant portion of patients could benefit from UKA over TKA, which is currently performed in over 88% of cases [51-53]. While revision rates need consideration for cost-effectiveness, recent advancements have yielded impressive UKA survival rates of 94% to 100% at 10 years and 95% at 15 years [54-57]. One study reported over 90% implant survival at 10 years, even for patients younger than 60 [58]. Large registry analyses have also indicated better pre- and post-revision quality of life for UKA patients than TKA patients [24]. These improvements in UKA prostheses and their longevity address concerns related to future conversions to TKA, enabling prompt patient discharge, reducing hospital costs, and effectively alleviating osteoarthritis symptoms. However, it should be noted that while single-centre studies highlight superior outcomes with UKA [59], low-volume surgeons experience significantly higher revision rates, emphasising the benefits of specialised high-volume centres for day-case UKA [60].

The study's limitations include a disproportionate number of THAs and TKAs compared to UKAs, reflecting the underrepresentation of UKAs in the NHS [19]. The small number of patients that achieved 24-hour discharge is representative of national averages within the NHS and is expected given the current median length of stay for THAs (3.2 days) and TKAs (3.3 days) [9], and hence is still generalizable to countries with similar low percentages of patients achieving 24-hour discharge. Our methodology did not incorporate patient-reported outcome measures, widely recognised for gauging clinical interventions' effectiveness from the patient's viewpoint [61]. These measures are critical in evaluating interventions. Due to surgeon preference, we could not include a uniform approach for THA procedures, with 75% of cases utilising a modified anterolateral and 25% utilising a posterior approach. There was no uniform opioid sparring anaesthetic policy available in the author's trust at the time of data collection. However, this research has proved pivotal in implementing a uniform anaesthetic protocol within the author's trust. Lastly, during comorbidity analysis, the hospital's electronic patient records did not distinguish between visual and hearing impairments, two separate variables for the FCI, leading us to consider them as a single variable.

## Conclusions

Our research identifies age as a significant factor influencing the success of same-day discharge after elective lower limb arthroplasties. Specifically, patients younger than 65 years undergoing UKA can often be discharged on the same day with minimal complications. Our findings suggest that complex scoring systems designed to predict same-day discharge might be unnecessary. Instead, age alone could be a straightforward criterion for deciding patient discharge, potentially simplifying the process when comorbidities are not a factor. However, the generalizability of these results is limited due to the small number of UKA cases in our study cohort. Additionally, there is conflicting evidence regarding postoperative complications and the feasibility of outpatient arthroplasty, especially in patients with pre-existing cardiopulmonary conditions or those who are in a poor functional state before surgery. These considerations warrant a cautious interpretation of our findings.
